# Levosimendan is superior to epinephrine on coronary flow for lipid-base resuscitation of bupivacaine-induced asystole in the isolated rat heart

**DOI:** 10.1186/s12871-018-0627-0

**Published:** 2018-11-20

**Authors:** Hongfei Chen, Fangfang Xia, Zhousheng Jin, Kejian Shi, Yun Xia, Le Liu, Thomas J. Papadimos, Xuzhong Xu, Limei Chen

**Affiliations:** 1 Department of Anesthesiology, The First Affiliated Hospital of Wenzhou Medical University, Shangcai village, Nanbaixiang town, Ouhai District, Wenzhou City, 325000 Zhejiang Province China; 2 Department of Anesthesiology, The Ohio State University Medical Center, Columbus, OH USA; 3 Department of Anesthesiology, The University of Toledo College of Medicine and Life Sciences, Toledo, OH USA

**Keywords:** Levosimendan, Epinephrine, Bupivacaine, Cardiotoxicity, Asystole

## Abstract

**Background:**

Successful resuscitation from asystole induced by bupivacaine requires the reestablishment of a sufficient coronary flow (CF) quickly. This study was designed to test whether levosimendan was superior to epinephrine in the reestablishment of crucial coronary flows after bupivacaine-induced asystole.

**Methods:**

The isolated, perfused, nonrecirculating, Langendorff rat heart preparation was used. Bupivacaine 100 μmol/L was perfused into rat hearts to induce asystole, and then for 3 min thereafter. Three experimental groups were assessed after asystole with infusions as follow: (1) a mixture of 2% lipid emulsion and 40 μmol/L bupivacaine (control group), (2) a mixture of 0.15 μg/mL epinephrine combined with 2% lipid emulsion and 40 μmol/L bupivacaine (epinephrine group), and (3) a mixture of 5 μmol/L levosimendan combined with a 2% lipid emulsion and 40 μmol/L bupivacaine mixture (levosimendan group). Coronary flow (CF), the time to recovery (T_recovery_), the number of ventricular arrhythmias, and cardiac function parameters were recorded for 40 min after heartbeat recovery.

**Results:**

All hearts in the control, epinephrine and levosimendan groups had heartbeat recovery. The rank order of the mean CF from highest to lowest was the levosimendan group > the epinepgrine group > the control group (*P* < 0.05). The rank order of T_recovery_ from shortest to longest was the levosimendan group < the epinephrine group < the control group (*P* < 0.01). During the recovery phase, isolated rat hearts developed more ventricular arrhythmias in the epinephrine group than in the levosimendan group (*P* = 0.01).

**Conclusion:**

Levosimendan is superior to epinephrine in producing higher CFs and faster recovery when reversing bupivacaine-induced asystole in the isolated rat hearts.

## Background

Bupivacaine is renowned for its long-lasting, high-quality anesthesia and analgesia. Unfortunately, it also may cause severe cardiotoxicity by inhibiting the sodium currents [1, 2], calcium currents [3], voltage-dependent potassium currents [4], and voltage-independent potassium currents in cardiomyocytes [5], as well as interfering with myocardial energy metabolism [6, 7]. Due to a variety of complex cytotoxic mechanisms, there is no anti-arrhythmic, vasopressor, or inotropic drug to effectively combat local anesthetic (LA)-induced asystole.

Intravenous lipid emulsions have been demonstrated to be effective in reversing LA-induced asystole in the guideline by American Society of Regional Anesthesia and Pain Medicine(ASRA) [8]. Epinephrine is part of the standard Advance Cardiac Life Support protocol in case of asystole for any causes, including LA-induced asystole. However, experimental data show that epinephrine may lead to ventricular fibrillation, aggravation of local anesthetic-induced arrhythmias [9], and increase the severity of cardiac dysfunction after resuscitation [10]. Moreover, Hiller et al. showed that resuscitation with epinephrine > 10 μg/kg resulted in severe pulmonary edema in rats, thus was detrimental to successful resuscitation outcomes [11]. Gavin et al. also reported that the use of epinephrine resulted in higher rate of severe neurologic impairment in adults with out-of-hospital cardiac arrest [12], thus the use of epinephrine is still controversial. Therefore, guideline for managing local anesthetic systemic toxicity now discourage the use of high doses of epinephrine during provision of care during adult advanced cardiovascular life support [8].

Levosimendan is a novel calcium sensitizer that enhances cardiomyocyte contractility without causing arrhythmias. Clinically, levosimendan is often used to improve cardiac function in patients with heart failure. Sebastian et al. [13] reported that levosimendan could effectively reverse the cardiac function caused by ropivacaine in the isolated guinea pig heart. Other laboratory studies also showed that levosimendan has a better therapeutic outcome in the treatment of local anesthetic toxicity [13–15]. Gruhn N et al. [16] found that levosimendan has a direct vasodilator effect on isolated porcine coronary arteries. This coronary artery dilatory effect of levosimendan may be beneficial for the effective removal of local anesthetics from cardiac tissue.

Thus, we conducted a prospective randomized animal study to investigate whether levosimendan was superior to epinephrine in increasing coronary flows (CFs) in reversing bupivacaine-induced asystole. In this study, we first established the best dose of levosimendan and then compared it to epinephrine and control groups.

## Methods

### Animals

Thirty-nine adult male Sprague-Dawley rats (SYXK 2015–0150) weighing between 280--310 g, were provided by Animal Centrer of Wenzhou Medical University. All animal protocols were approved by the Animal Care and Use Committee of Wenzhou Medical University (wydw2015–0121, Zhejiang, China).

### Drugs

Bupivacaine (bupivacaine hydrochloride, Hefeng Pharmaceutical Co., Ltd., Shanghai, China), lipid (20% Intralipid, Huirui Pharmaceutical Co., Ltd., Suzhou, China), epinephrine (epinephrine hydrochloride, Hefeng Pharmaceutical Co., Ltd., Shanghai, China), and levosimendan (levosimentan Injection, Qilu pharmaceutical Co., Ltd., Jinan, China) were used.

### Random table method

(1) Starting from any number in the random number table, obtain a random number of experimental units in order from the unified direction. (2) Divide the non-repeating random number by the number of groups to obtain the remainder. (3) Arrange the rats into groups according to the remainders. (4) If the numbers in each group are different, we would choose any one from a plurality of groups, and the remainder obtained by dividing the random number by the group and arrange it again.

### Preparation of isolated hearts

The isolated, perfused, nonrecirculating, Langendorff rat heart preparation was used in our study, as described previously [17]. In brief, rats were anesthetized by the intraperitoneal injection of 350 mg/kg chloral hydrate, and 1000 U/kg heparin was administered to prevent the formation of intracoronary microthrombi. Hearts were rapidly excised after euthanasia by decapitation and perfused via the coronary arteries by catenating the aorta to a cannula (ML870B2, AD Instruments, Australia). The constant perfusion pressure was 120 mmHg, and a modified Krebs-Henseleit buffer (KHB) was used and is described as follows: NaCl 118 mmol/L, KCl 4.7 mmol/L, MgSO_4_ 1.2 mmol/L, KH_2_PO_4_ 1.2 mmol/L, NaHCO_3_ 25.0 mmol/L, CaCl_2_ 2.5 mmol/L, and glucose 10 mmol/L. The solution was exposed to 95% O_2_ and 5% CO_2_, and pH was maintained at 7.40 ± 0.05. All elements of the perfusion apparatus were water-jacketed and maintained at 37 °C. The left ventricular pressure was continuously monitored by a latex balloon placed in the left ventricle. Saline was intermittently injected into the balloon to maintain the left ventricular end-diastolic pressure at 4–10 mmHg.

Hemodynamic variables and derivatives (coronary flow (CF), heart rate (HR), left ventricular developed pressure (LVdevP = systolic pressure- diastolic pressure), rate-pressure product (RPP = HR × LVdevP), and maximum change rate of left ventricular pressure increase and decrease (+dP/dt_max_) were collected using a PowerLab biological signal processing and analysis system (ML870, Australia Ad Instruments) and the Chart 5.5.6 biological signal recording software. Electrocardiography (ECG) electrodes were consistently placed in a “leadII” position: one epicardial electrode was placed the right atrium and a second epicardial lead was placed at the apex of the heart. The experimental protocol was started when CF, HR, RPP and + dP/dt_max_ had reached steady-state baseline conditions, which was 25 min after artificial perfusion had commenced.

### Experimental protocol

#### Part 1-----decision of the optimal levosimendan concentration in the lipid-based reversal of bupivacaine-induced asystole in the isolated rat heart

Accordingly, a prospective randomized animal study was undertaken. Fifteen hearts were isolated and randomly allocated by the random table method before the study into three group, with 5 hearts per group as follows: levo2.5 group, levo5 group and levo10 group. After reaching a steady state, bupivacaine was perfused into the hearts to cause asystole, and then 3 min thereafter. They were then perfused with 40 μmol/L bupivacaine along with 2% lipid emulsion and levosimendan at concentrations of 2.5 μmol/L, 5 μmol/L or 10 μmol/L,respectively. All groups were perfused for 40 min after cardiac recovery. Heartbeat recovery was defined as an unassisted regular rhythm with an RPP > 10% of the baseline for > 1 min.

#### Part 2-----comparison among the control group, epinephrine group and levosimendan group

**Fig. 1:**
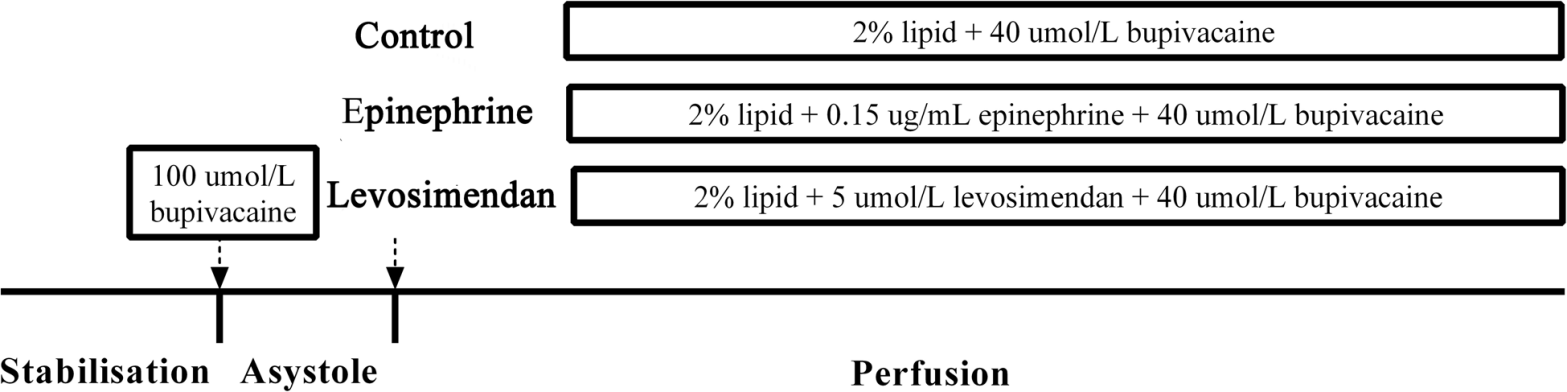
Flowchart of the experiment. Notes: All groups were perfused for 40 min after cardiac recovery. Abbreviations: Control group: lipid only; Epinephrine group: combined epinephrine + lipid; Levosimendan group: combined levosimendan + lipid. Ts:the time from perfusing 100 μmol/L bupivacaine to rebeat

Twenty-four hearts were isolated and randomly allocated by the random table method before the study into three group, with 8 hearts per group (Fig. 1) as follows: control group, epinephrine group and levosimendan group. Group assignment was randomized with investigators blinded to group assignments. After stabilization, 100 μmol/L bupivacaine was perfused into the hearts until asystole occurred and then for 3 min thereafter. The experimental perfusion was then started according to the assigned group: a 2% lipid emulsion and 40 μmol/L bupivacaine mixture was then perfused in the control group; 0.15 μg/mL epinephrine combined with 2% lipid emulsion and 40 μmol/L bupivacaine mixture in the epinephrine group; and 5 μmol/L levosimendan combined with 2% lipid emulsion and 40 μmol/L bupivacaine mixture in the levosimendan group. All groups were perfused for 40 min after cardiac recovery.

We compared the time from initiation infusion of bupivacaine to asystole (designated as T_asystole_) and the time from the finish of the 100 μmol/L bupivacaine infusion to cardiac recovery (designated as T_recovery_) in three groups. The cardiac function parameters (HR, RPP and + dP/dt_max_) were recorded or calculated. The parameters were recorded at baseline (T_baseline_), and at 1, 5, 10, 15, 20, 25, 30, 35 and 40 min after heartbeat recovery. The ECG was monitored throughout the experiment and the duration of ventricular arrhythmia was recorded during the 40-min post recovery perfusion. In this study, we defined ventricular tachycardia, flutter or fibrillation for 3 s or more as a ventricular arrhythmia.

### Statistical analysis

The determination of the number of animals in each group was based in our preliminary study (3 rats in each of 3 group), in which the CF were 11.1 ± 1.0, 14.0 ± 0.9, and 16.7 ± 0.6 in control, epinephrine and levosimendan groups, respectively. Using a two-tailed type one error of 5% and type two error at 10% (= 0.05, β = 0.1), the sample size of 8 per group was obtained by Power Analysis and Sample Size (PASS; 11.0). We enrolled 8 rats per group.

SPSS (version 19.0, Chicago, IL) was used to carry out the computations. Data were tested for normality using the Shapiro-Wilk test; normally distributed data were presented as the means ± standard deviation. Weights, T_asystole_ and T_recovery_ were analyzed by one-way ANOVA, and then the LSD test was used between two groups when significance was achieved. Continuous cardiac function parameters among groups were compared using repeated-measures of analysis of variance. Statistical significance was considered as *P* < 0.05. The incidence rate of ventricular arrhythmia among the groups was compared with the Fisher’s exact test with Bonferroni correction post-testing when significance was achieved (*P* < 0.05). Statistical significance was considered as *P* < 0.017.

## Results

### Determination of the optimal concentration of levosimendan in lipid-based reversal of bupivacaine-induced asystole in the isolated rat heart

After reaching steady state, there was no statistically significant difference in CF, HR, RPP, +dP/dt_max_ and T_asystole_.

**Fig. 2:**
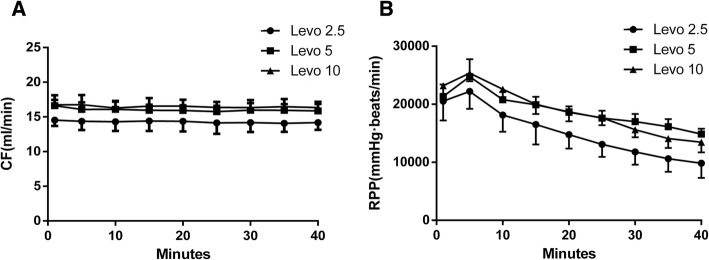
Coronary flows (CFs), Rate-pressure products (RPPs) in levo2.5, levo2.5, and levo2.5 groups, are shown during recovery from bupivacaine-induced asystole (mean ± standard deviation; *n* = 5 for all values). Notes: Figure 2a, Coronary flows (CFs), Levo5 vs. Levo2.5, *P* = 0.023; Levo10 vs. Levo2.5, *P* = 0.006; Levo5 vs. Levo 10, *P* = 0.496. Figure 2b, Rate-pressure products (RPPs), Levo5 vs. Levo2.5, *P* = 0.006; Levo10 vs. Levo2.5, *P* = 0.005; Levo5 vs. Levo10, *P* = 0.905. Abbreviations: Levo2.5: levosimendan injection at concentrations of 2.5 μmol/L; Levo5: levosimendan injection at concentrations of 5 μmol/L; Levo10: levosimendan injection at concentrations of 10 μmol/L.

All three groups demonstrated heartbeat recovery. There were no differences in mean CF and mean RPP between the levo5 and levo10 groups, but both were significantly higher than those in the levo2.5 group (Fig. 2a and b). There was no difference in T_recovery_ and CF between Levo5 and Levo10 groups (20.6 ± 2.7 s, 20.9 ± 3 s), but both were significantly shorter than those of levo2.5 group (25.6 ± 3.7 s).

#### Baseline values and weight of rats

Baseline values of weight and cardiac function are detailed in Table 1. No statistically significant differences were observed in CF, HR, RPP, +dP/dt_max_, T_asystole_ and rat weight at the beginning of the experiment (Table 1).

**Table 1:** Baseline Values of Key Variables and weight for control, epinphrine and levosimendan groups

	Control	Epinephrine	Levosimendan	*P*
Weight(g)	289 ± 10	287 ± 6	288 ± 6	0.940
HR(beats/min)	287 ± 10	285 ± 10	285 ± 6	0.913
LvdevP(mm Hg)	140 ± 10	139 ± 10	137 ± 6	0.768
RPP(mm Hg·beats/min)	40,183 ± 2279	39,461 ± 2073	39,101 ± 2092	0.599
+dP/dt_max_ (mm Hg/s)	4152 ± 358	4189 ± 214	4141 ± 248	0.704
CF(ml/min)	20.5 ± 0.9	21.3 ± 0.9	21.4 ± 0.7	0.089
Ts(s)	33 ± 6	36 ± 11	34 ± 8	0.188

Values are given as the mean ± standard deviation, n = 8 in each group. Baseline values for major variables showed no significant differences among the 3 groups. Control: lipid only; Epinephrine: combined epinephrine+ lipid; Levosimendan: combined levosimendan + lipid. HR: heart rate; LVdevP: left ventricular developed pressure; RPP: rate-pressure product; +dP/dt_max_: maximum change rate of left ventricular pressure increase and decrease; CF: coronary flow; T_asystole_: the time from initiation to asystole

#### Coronary flow (CF)

**Fig. 3:**
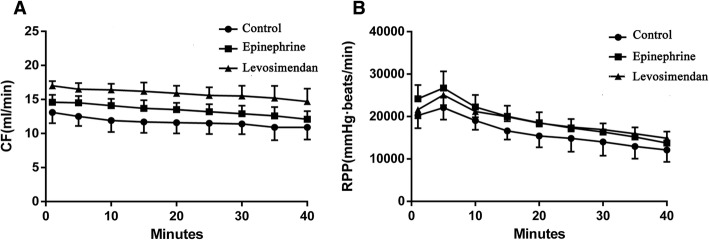
Coronary flows (CFs), Rate-pressure products (RPPs) in control, epinephrine and levosimendan groups are shown during recovery from bupivacaine-induced asystole (mean ± standard deviation; *n* = 8 in each group). Notes: Figure 3a, Coronary flows (CFs): Levosimendan group vs. Control group, *P* < 0.001; Epinephrine group vs. Control group, *P* = 0.038; Levosimendan group vs. Epinephrine group, *P* = 0.004. Figure 3b, Rate-pressure products (RPPs): Levosimendan group vs. Control group, *P* = 0.027; Epinephrine group vs. Control group, *P* = 0.013; Levosimendan group vs. Epinephrine group, *P* = 0.946. Abbreviations: Control group: lipid only; Epinephrine group: combined epinephrine + lipid; Levosimendan group: combined levosimendan + lipid

After heartbeat recovery, the mean CF at 40 min in the levosimendan group was significantly higher than that of the other two groups (levosimendan versus control, *P* < 0.001; Levosimendan versus epinephrine *P* < 0.01). The mean CF in the epinephrine group was higher than that of the control group (*P* < 0.05) (Fig. 3a).

#### Time to recovery (T_recovery_)

**Fig. 4:**
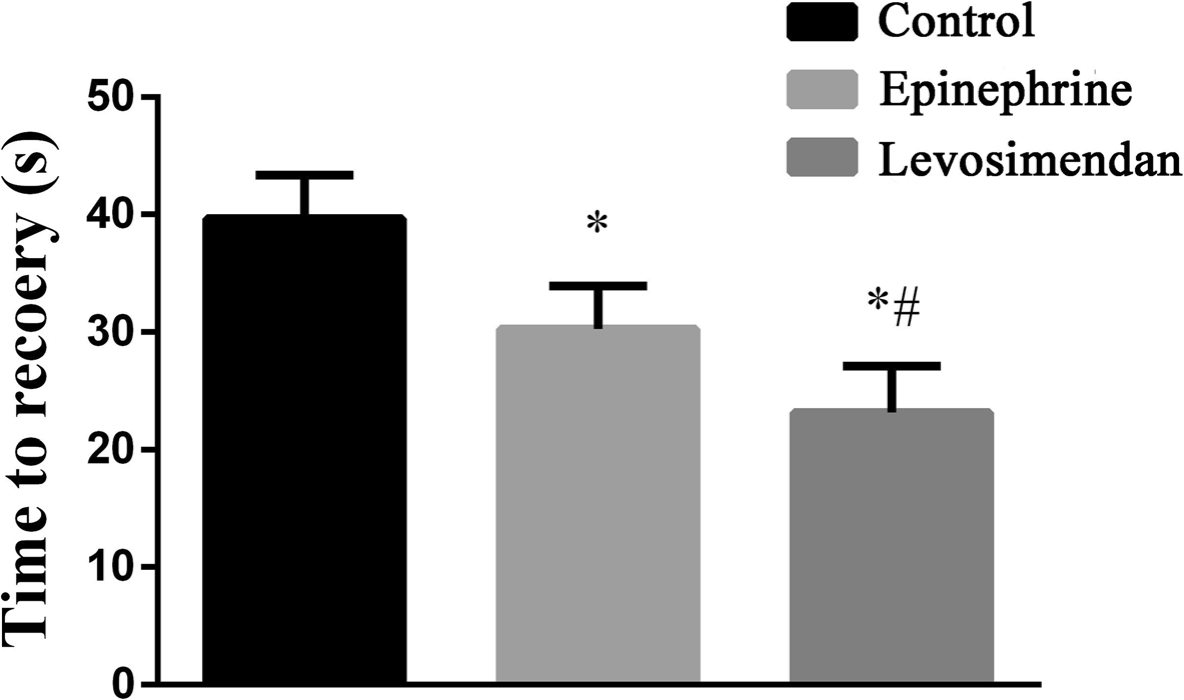
Time to recovery from asystole induced by bupivacaine in control, epinephrine and levosimendan groups (*n* = 8 for all values). Notes: ^*^P < 0.05 vs. Control group; ^#^*P* < 0.05 vs. Epinephrine group. Abbreviations: Control group: lipid only; Epinephrine group: combined epinephrine + lipid; Levosimendan group: combined levosimendan + lipid

All hearts in the control, epinephrine and levosimendan groups had heartbeat recovery. T_recovery_ in levosimendan group was significantly shorter than that of the other two groups (levosimendan versus control, *P* < 0.001 and levosimendan versus epinephrine, *P* = 0.001). T_recovery_ in the epinephrine group was shorter than that of the control group (epinephrine versus control, *P* < 0.001) (Fig. 4).

#### Incidence rate of ventricular arrhythmia

The incidence of ventricular arrhythmias in the epinephrine group (7/8, 87.5%) was significantly higher than that in the levosimendan group (1/8, 12.5%, *P* = 0.01) and in the control group (1/8, *P* = 0.01).

### Cardiac function variables

#### Heart rate

During the 40-min recovery phase, the mean HR in the levosimendan and epinephrine groups are significantly higher than the mean HR in the control group (levosimendan versus control, *P* = 0.01 and epinephrine versus control, *P* = 0.001). There was no significant difference in HR between epinephrine and levosimendan groups (*P* = 0.735).

#### Rate-pressure product

During the 40 min after heartbeat recovery, the mean RPP values in the epinephrine and levosimendan groups were increased significantly as compared to the control group (levosimendan versus control, *P* = 0.027; epinephrine versus control, *P* = 0.013). There was no significant difference in RPP between the epinephrine and levosimendan groups (*P* > 0.05, Fig. 3b).

#### Maximum change rate of left ventricular pressure increase

During the 40 min recovery phase, the mean + dP/dt_max_ values in the epinephrine and levosimendan groups were significantly higher the value for the control group (*P* < 0.05). There was no significant difference in +dP/dt_max_ between the epinephrine and levosimendan groups (*P* > 0.05).

## Discussion

This study showed that in the bupivacaine-induced asystole in the isolated rat model, the levosimendan group had significantly higher mean CF and shorter T_recovery_ than the epinephrine and control groups. The levosimendan group was also found to induce fewer ventricular arrhythmias than the epinephrine group in the isolated rat heart. Feldmanet al. [18] found that bupivacaine achieves its highest concentrations in blood and myocardium within seconds after intravenous injection. It then declines rapidly over 3–5 min, but continues to remain in low concentrations in the blood and myocardium. To model the slow elimination of bupivacaine from the plasma, we continuously perfused 40 μmol/L bupivacaine in each group as a background drug concentration [18]. As suggested by Liu et al. [19], we chose 0.15 μg/ml as the optimal concentration of the epinephrine. Based on the results of the first part of this study, we found the 5 and 10 umol/L of levosimendan have no difference in improving CF and RPP. Since high concentration might produce a higher risk of some side effects, we chose a lower concentration of 5 umol/L to produce the fast and effective recovery of myocardial function.

We observed that levosimendan increased the coronary blood flow more than epinephrine in our isolated heart model. It is of interest that both Levosimendan and epinephrine can dilate arteries, but by different mechanisms. Stehr et al. [13], demonstrated that levosimendan significantly reversed the ropivacaine-induced reduction in coronary blood flow (in the Langendorff heart preparation/model) through activation of ATP-sensitive K channels [20]. On the other hand, epinephrine, unlike levosimendan, has multiple effects on coronary arteries including constriction (via α-receptors) and vasodilation (via β-receptors) [21]. These β-receptors work via cyclic adenosine phosphate (cAMP), but the effect of cAMP is limited in the presence of local anesthetics. Thus, we propose that levosimendan is better at vasodilation of coronary arteries than epinephrine in the presence of bupivacaine.

Recently, Liu [19] reported that in the bupivacaine-induced isolated rat heart model, the combination of epinephrine and lipid emulsions resulted in better cardiac function and coronary blood flow than lipid emulsions alone. Our study found that levosimendan combined with a lipid emulsion enhanced the CF, which resulted in faster recovery in the isolated rat hearts than did epinephrine combined with lipids or lipids alone after asystole. This may be related to the dilation of coronary arteries by levosimendan, which may enhance the elimination of bupivacaine.

Additionally, epinephrine may induce arrhythmias and aggravate myocardial oxygen demand through a rise in intracellular calcium [22, 23]. However, levosimendan enhances cardiac function without increasing intracellular calcium concentration, which may the reason for the decreased occurrence of ventricular arrhythmias with its use [13]. In this study, 7/8 (87.5%) of epinephrine group developed ventricular arrhythmias, whereas only 1/8 (12.5%) of in the levosimendan and control groups had ventricular arrhythmias, respectively. It was not difficult to surmise that levosimendan is more advantageous in this respect.

As a calcium sensitizer, although studies have shown that levosimendan does not improve the mortality in patients after cardiac surgery [24] and sepsis [25], it does improve heart function after cardiac surgery [26] and sepsis [25, 27]. Different with those chronic circulatory failure process, the model of LA-induced asystole is an acute circulatory failure process, and the timely treatment of levosimendan by improving CF and RPP is extremely important for the prognosis of patients. So far, there has been little research on the treatment of local anesthetic poisoning with levosimendan. It is unclear who has more advantages in levosimendan and epinephrine.

Aittomaki et al. [13] reported that levosimendam was more effective than saline in restoring heart rate and blood pressure inhibited by bupivacaine in the swine experiment than saline. Recently, Gokahmetoglu et al. [15] reported that the combination of levosimendan and lipid was more efficacious than lipid alone when treating bupivacaine-induced cardiac arrest in rabbits. These studies demonstrated the effectiveness of levosimendan in the treatment of local anesthetic toxicity without an explanation of the specific mechanism. We speculate that the vasodilation of coronary arteries may be an important mechanistic advantage of levosimendan in the treatment of local anesthetic toxicity, this remains to be determined.

There are limitations to our study. In this model, we could not determine the effect of epinephrine and levosimendan on systemic vascular smooth muscle cells, which might have a significant impact on blood pressure. Furthermore, adequate oxygen and normal internal environment were provided to the hearts during the Langendorff perfusion in our study while hypoxia and acidosis were frequently appeared in local anesthetic-induced asystole. Therefore, we need to explore the therapeutic mechanism of levosimendan and epinephrine on local anesthetic toxicity via imitating the environment of cardiac hypoxia and acidosis in the further study.

## Conclusion

In conclusion, our results indicate that 5 μmol/L of levosimendan combined with 2% lipid emulsion improved CF more than 0.15 μg/mL epinephrine combined with 2% lipid emulsion. This leads to faster recovery from bupivacaine-induced asystole in the isolated rat heart.

## Abbreviations

+dP/dtmax: maximum change rate of left ventricular pressure increase and decrease
CFs: coronary flows
ECG: Electrocardiography
HR: heart rate
KHB: Krebs-Henseleit buffer
LVdevP: left ventricular developed pressure
RPP: rate-pressure product
Trecovery: time to recovery
